# Edema

**Published:** 1899-10

**Authors:** Stephen Y. Howell

**Affiliations:** Buffalo, N. Y.


### EDEMA.

By STEPHEN Y. HOWELL, A. M., M. D., M. R. C. S., Eng.
Buffalo, N. Y.

CONTEMPLATION of the structural details of the human body, nature's apparent *chef-d'œuvre*, must be attended with wonderment that the frail machine should, in any case, survive the manifold dangers which threaten its existence, that it should fulfil the expectations of its creator. His minute morphology shows man to be a multicellular animal with stromal supports of various textures and designs, so combined and endowed as to perform with certainty the manifold functions which are essential to his well-being and which enable him to work out his destiny.

These myriads of component cells, massed in collaborating organs, the latter each with special functional endowments, are most marvelously and wisely provided for. To furnish the frail little workers with the vital energy which shall enable them to perform their allotted tasks, provision is made for their food-supply, for their growth, multiplication and decay, for the removal of their waste, and, finally, in the case of the glandular organs, for the further use in the economy, or the final expulsion therefrom, of their special products, in the form of secretions and excretions.

To properly realise the feasibility of this close attention to the wants of the individual cell it is only necessary to remember that the socalled solid tissues are solid in name only. Even the bones, to which the above epithet is most fittingly applied, are fairly riddled with channels, from the large Haversian canals to the microscopic canaliculi, through which access is afforded to each individual cell, snugly ensconced within its stromal investiture.

In like manner the cells of the softer tissues are everywhere accessible through the medium of branching and inoculating ways, varying in size from those readily appreciable to the naked eye down to the delicate plexiform network of clefts or spaces which are in immediate contact with the cells and are only in part supplied with a delicate endothelial lining. Thus it is that provision is made for the cell-wants through this elaborate system of approaches, in which flow the fluid tissues, the blood and lymph. When food has been converted by the digestive processes into materials capable of assimilation by the cells, these nutritive compounds are transferred to the blood through the veins and lacteals. From this blood there normally exudes, through the walls of the small veins and capillaries, a fluid rich in dissolved or suspended food-stuffs, termed lymph, which enters the lymphatic capillaries and is by them conveyed to the cells. Thus carbohydrates, fat, albumin, oxygen, salines and water compose the elaborate bill-of-fare presented to the ultimate workers of the body; while the excretory organs discharge urea and other proteids, carbonic acid and water. It will be remarked that these excreta are simpler compounds, chemically speaking, than the ingesta, the difference in the heat values of the two being appropriated by the tissues and stored up as potential energy or force, to be converted into kinetic energy as occasion may demand. The special products of the cells are conveyed away through secretory ducts, while the diverse excreta are deposited in the surrounding plasma stream to be returned to the blood indirectly through the lymph ducts, or directly by endosmosis. Normally, then, the tissues are constantly traversed by tiny streams of fluid, exuding from the bloodvessels to be again gathered up by the lymph-drains.

Associated with the lymphatic vessels, and a part of the same system, are the serous cavities of the body, which are in reality enormous sacs, whose surfaces are in communication with the lymph-drains through the medium of minute intercellular stomata. Thus the peritoneal cavity, pleura, pericardium, tunica vaginalis testis, joint cavities, ventricles and subarachnoid space, aqueous chambers of the eye and the aural labyrinths are all supplied with lymph in quantities suitable for the proper fulfillment of their various functions, any excess being promptly drained off. When for any reason, however, the fluid transudations from the bloodvessels are excessive and the drainage system proves inadequate to cope with the supply, the tissues become saturated to a pathological degree and fluid accumulates in the body-cavities. To describe such a condition the terms "dropsy," "hydrops" or "general dropsy" are employed, meaning, to repeat, an accumulation of fluid transudate from the bloodvessels in the interstices of the connective tissue, in the serous cavities of the body, or in both. When the dropsical effusions are limited to the skin and subcutaneous tissue and are extensive in area the condition is known as "anasarca;" while edema describes a dropsical condition of the integumentary structures limited in area or confined to the connective tissue of organs.

Thus we speak of edema or a dropsical condition of the eyelids or ankle, not anascarca of these structures; of edema of the lungs, liver, kidney and similar states.

Other local or regional dropsies are known as "ascites," an effusion limited to the peritoneal cavity; "hydrothorax," to the pleural spaces; "hydropericardium," to the pericardial sac. By "hydrocephalus," internal and external, we mean dropsy of the ventricles or subarachnoid and subdural spaces respectively; by "hydrocele," dropsy of the peritoneal extension which persists as the tunica vaginalis testis, and the like.

The fluid in dropsical conditions is always derived from the blood-vessels, but varies greatly in composition. As a rule, it is of lower specific gravity than the blood plasma or liquor sanguinis, contains less albumin, and has a slight tendency to coagulate, because of its comparative poverty in the fibrin-factors.

The effusions which characterise edema and anasarca are usually the most watery, while those which accumulate in the pleura, peritoneum and tunica vaginalis are rich in albumin. As we shall see, however, in certain conditions the pure blood itself seems to leave the vessels.

*Causes.*—The various pathological conditions resulting in edema may be classified in four groups: (1) the edema of engorgement; (2) inflammatory edema; (3) the hydremic variety, and (4) the angioneurotic of Jacob.

Under the first head may be arranged all those causes resulting in obstruction to the return circulation in the veins and capillaries, such as obtain in mechanical, venous, passive, or obstructive hyperemia; in cases of feeble cardiac impulsion or diminished vis-a-tergo and in lymphatic obstruction. Because of the free anastomoses of the venous system, the obstruction of a single vein is unlikely to cause local dropsy, the probability of this event being in inverse proportion to the existing collateral circulation. Gravity, too, is a factor of which sight must not be lost. Its effects are most apparent in the lower extremities, when the patient stands or walks, whereby the pressure upon the ill-nourished veins and capillaries is greatly increased and leakage is favored. Familiar examples of this are met with in ambulatory cases of hydremia or cardiac disease, in whom swelling of the legs is observed during the day, only to abate at night when the recumbent position is assumed.

If a single lymph duct be obstructed the area drained by it may become edematous when collateral lymphatics or veins fail to assume its burden. Should the thoracic duct itself be alone involved general dropsy does not necessarily ensue, though ascites or hydrothorax may result. When its obstruction is associated with tricuspid insufficiency, however, general serous exudation is greatly favored.

Examples of obstruction to the return circulation are very frequently met with by the physician. A moderately tight finger-ring, for instance, which does not materially impede the arterial supply but checks the venous and lymphatic outflow, is the cause of edema of the constricted member. If not removed the added pressure of the effused serum may cut off the arterial supply entirely, when gangrene will ensue. Interference with the portal circulation in consequence of hepatic cirrhosis or the pressure of a carcinoma favors ascites. Pulmonary congestion, the result of mitral disease, is attended with edema of the lungs. Obstruction to the systemic circulation wrought by tricuspid lesions, the latter either primary, or secondary to mitral defects; pressure upon the iliac veins by the gravid uterus; the venous thrombosis resulting in milk-leg; diminished muscular contractions in the invalid or bed-ridden patient; valvular incompetence in the veins of the extremities, which results from dilatation; lessened thoracic aspiration in pleural disease, or the increased obstructive pressure within the thorax induced by the playing of wind instruments, all—have a causal influence. The vis-a-tergo, or driving force of the circulation, the diminution of which is attended with venous hyperemia and edema, is especially effected in chronic exhausting diseases, in the acute fevers such as typhoid, in degenerative changes resulting in dilatation, and the like, all of which lessen the motor power of the heart and tend to cause its failure. The driving power of individual arteries likewise may be lessened by direct obstructions from without, or by reason of changes in the walls, wrought by the fatty, atheromatous or fibroid degenerations.

*Results.*—The ultimate result of the hyperemia induced by these various causes is impaired vascular nutrition. The walls of the veins and capillaries suffer from interference with their food-supply, and the degree of the consequential effects varies directly with the extent of said nutritive interference and with the intravascular pressure.

### INFLAMMATORY EDEMA.

For our present knowledge regarding the phenoma of inflammation we are largely indebted to the elaborate and extended experiments of Cohnheim and his school. The whole subject is one of vital import and pregnant with interest, but I shall not weary you with a close résumé of the various steps by which these observers were led to their final. conclusions. Let it suffice if I simply recall those points which pertain more immediately to the subject under discussion. If we watch the exposed mesentery of a frog, for example, the first step in the process of inflammation is noticed in the dilatation of the arteries, followed by the enlargement of the veins and capillaries. At first and for a short space of time, this increase of lumen area is attended with a greater rapidity of the blood-currents, but soon the fact that the blood courses more and more slowly through the dilated vessels becomes very noticeable. Finally, the primal causes persisting, all onward blood movement in the capillaries ceases, the mass merely swaying to and fro rhythmically with the pulse—the so-called stage of oscillation. Succeeding this is a complete stasis of the stream, resulting in the death of the capillary walls whose food supply has thus been cut off, when thrombosis finally ensues. In all these cases of denutrition of the vascular endothelia, where death has not resulted from their lengthened fast, the vitality of the vessel-wall is restored by again feeding the cells; that is, by reëstablishing the blood stream in the affected vessels. The cure results from the *vis medicatrix naturæ.*

Early in the course of these vascular and circulatory changes the inflamed area will be seen to swell. The normal fluid transudate increases greatly in quantity, while its quality is likewise altered. The lymphatics being unable to drain off the swelling flood which permeates the tissues, it accumulates in the lymph spaces. Following quickly in the wake of the fluid are seen new cells; and should a small vein or a capillary in the inflamed region be watched, the escape or diapedesis of leucocytes through their walls will explain the genesis of the new arrivals. Later, and in proportion to the tissue-insult and vascularity, the red blood corpuscles will be observed to pass out of the capillaries as the blood-stream slows, both white and red cells being wafted farther into the tissues by the serous streams which accompany them. If the fluid transudate of inflammation be compared with that which exudes from the vessels in venous hyperemia an excess of albumin, phosphates and carbonates will be found in the former, together with an increased disposition to coagulate; that is, the resemblance to the liquor sanguinis becomes closer. As the inflammatory process increases in severity the escaping fluid resembles the blood itself more and more, till the two become finally identical in composition.

To understand the real *raison d'être* of these observed phenomena in hyperemia and inflammation was long the great desideratum of the professional world. The paramount importance of such knowledge was universally felt, since it would throw a flood of light into many of the dark places which the persistent efforts of the physiologist and pathologist had failed to illumine.

Aided by an efficient staff of able collaborators, and stimulated by his rediscovery of cell-migration from the bloodvessels in 1867, Cohnheim, after the long and arduous labors involved in innumerable experiments and deep thought, subsequently announced the theory which now meets with well-nigh universal acceptance. The essential lesion was declared to be a "molecular change" in the vessel wall, possibly chemical in character, since no alteration of structure could be detected. Whether the endothelial lining-cells of the vessels, the intercellular cement-substance or both were the structures involved in this change could not be definitely determined. Resulting from an injury, direct or indirect, which tended to devitalise the morphological constituents of the bloodvessels, this molecular change altered the porosity of the vessel walls and permitted the escape—first, of fluid of changing qualities, and, later, of blood-cells. Cohnheim likened this process to one of filtration, embodying his views in the pithy statement that "change of filter means change of filtrate."

### HYDREMIC, CACHECTIC OR UREMIC EDEMA.

Qualitative variations in the blood, resulting in its impoverishment, and which are properly comprised under the term spanemia, play a large part in the production of dropsy. Hydremia due to an actual (hydremic plethora) or relative excess of water in the blood and the toxemias resulting in malnutrition and nerve irritation have all a causal influence. Instances of these blood-conditions are seen in renal disease, anemia, chlorosis, malarial cachexia, scurvy, slow starvation, loss of blood, suppuration, chronic discharges, continued fever, growth of neoplasms, prolonged lactation and all debilitating conditions resulting in hydremia.

Cachectic edema frequently occurs in feeble and poorly nourished individuals; and one of the commonest forms of dropsy is the slight edema of the legs in anemic persons whose heart and lungs are free from apparent disease. This may be attributed to various factors: hydremia, blood pressure, lessened nutrition of vessels, and, perhaps a tendency to vasomotor paresis.

Fourth, the influence of the nervous system in the production of dropsy must not be lost sight of, the so-called angioneurotic class of Jacob.

Paralysis of the vasomotor fibers, either central or peripheral, or stimulation of the vasodilator nerves, may result in hyperemia with transudation and ecchymoses in the areas affected, as the lungs, brain, pleura, intestines and kidneys. Serous apoplexy may thus be accounted for.

In paralysis the affected limbs sometimes become edematous, especially in anterior poliomyelitis or infantile palsy. Edema of the face may occur in affections of the trifacial or 5th nerve. The irregular and anomalous distributions of the fluid transudates in general dropsies may be due to the local disturbances of innervation.

*Gross Appearances.*—The recognition of dropsical conditions is easy, as a rule. When the integumentary structures are involved the parts are swollen, particularly in situations where the subcutaneous connective tissue is abundant and lax, as in the eyelids, penis and scrotum. The skin-folds are apt to be obliterated, and stretching may be sufficiently marked to occasion the dermal cicatrices such as commonly follow pregnancy. The parts are slightly translucent, doughy to the touch and retain the impress of the finger-tip when pressure is made for a time, "pitting," as it is called. The color is pale, as a rule, in renal dropsy; while in heart disease the edematous lips, skin and mucous membranes may be dusky or cyanosed.

When edematous parts are incised, the tissues are seen to be thickened, jelly-like and translucent, or watery; while pressure extrudes a serous fluid which is abundant in lax connective tissues and loosely constructed organs like the lungs, but comparatively scanty in denser viscera, such as the kidney.

Microscopically, the dropsical part is found to be made up of a delicate reticulum of connective tissue, the spaces of which are filled with fluid and lined with flat endothelial cells, as a rule. When the large body cavities are involved the sensation of fluctuation on tapping with the fingers is readily elicited, particularly in ascites. Percussion and exploratory aspiration are further diagnostic aids which rarely fail us.

The differential diagnosis between the cardiac, renal and anemic forms of general dropsy is rarely attended with any great difficulty; but in those advanced cases where all three forms coexist it is often impossible to determine their precise order of sequence. Cardiac dropsy generally first shows itself in the feet and ankles; there is often a history of previous articular rheumatism in the case of young people, and physical exploration discloses heart trouble. In the renal variety we are aided by the urinary examination, this usually enlightening us not only as to the existence of kidney disease, but also disclosing its more precise nature; the edema first affects the eyelids in the majority of cases, while the external genitals are involved earlier and more frequently than in cardiac disease. It should be remembered, however, that the earliest observed symptoms may be convulsions and coma.

In hydremic or anemic dropsy the spanemia is easily determined by blood-counts and by the percentage of hemoglobin, in the absence of cardiac or renal lesions. The effusion, too, is not extensive, and is limited to the integumentary structures, generally of the face and ankles. Oftentimes the face is swollen in the morning, the feet in the evening.

*Prognosis.*—We are not to lose sight of the fact that dropsy is not a disease *per se*; and whether it be temporary or lasting, whether it merely contributes to the patient's discomfort or hastens his taking-off depends rather on the nature of the primal disease of which it is merely a symptom. Remove the cause and we cure the dropsy. In renal and cardiac disease, for example, large dropsical effusions are of evil omen since they herald the breaking-down of the patient's conservative forces and proclaim a waning vitality. On the other hand, in scarlatinal anasarca, the edema of pregnancy and similar acute forms, the outlook is favorable, as it is likewise in the nonpernicious forms of anemia.

*Treatment.*—The various remedial measures to be adopted may be conveniently arranged under three heads : (1) Efforts to remove the fluid accumulations. This may be accomplished by puncturing the affected tissues with round needles, by incisions in the most dependent parts, and by the use of the trocar or aspirator. Indirectly, their absorption is favored by measures which promote diaphoresis, by the use of diuretics and hydragogue cathartics. (2) The removal of all obstructions to the circulation in bloodvessels and lymphatics is indicated, whenever this is possible. (3) General measures should be adopted which will tend to restore and maintain the patient's strength and enhance the nutrition of his tissues. We can relieve pain, support the swollen parts with suitable bandages, see that proper clothing is worn, look after the diet, and exhibit such tonics as are indicated.

164 Franklin Street.

